# Some Personal Experiences in Gall-Bladder Surgery1Read at the Twenty-Second Annual meeting of the American Association of Obstetricians and Gynecologists at Fort Wayne, September 21-23, 1909.

**Published:** 1909-11

**Authors:** Herman E. Hayd

**Affiliations:** Buffalo, N. Y.


### BUFFALO MEDICAL JOURNAL.

VOL. LXV. NOVEMBER, 1909. NO. 4

### ORIGINAL COMMUNICATIONS.

### Some Personal Experiences in Gall-Bladder Surgery.[1]

BY HERMAN E. HAYD, M. D., M. R. C. S. Eng., Buffalo, N. Y.

*(From American Journal of Obstetrics)*

BILIARY drainage is exceedingly important in the treatment of the associated complications of cholecystitis and cholelithiasis, and is best accomplished through the gall-bladder. A few years ago many good surgeons and operators advocated the removal of the gall-bladder when this viscus was diseased, and in doing so they did not then realise that they were robbing the patient of the most powerful agent for the cure of his symptoms—namely, biliary drainage. At that time, in support of the idea that the gall-bladder was a useless appendage and was simply a reservoir for the storage of bile, there appeared numerous articles in the journals, written by biologists and comparative anatomists, (notably one by Woods Hutchinson in the *Medical Record,* May 16, 1903), by analogy contending that because certain animals had no gall-bladders, man could do without his, and this was especially plausible when it could be shown that a number of persons had had their gall-bladders removed and were in good health. Soon, however, the pathologist and clinician found that with acute and chronic gall-bladder disease there often existed secondary and dangerous complications, as cholangeitis and pericholangeitis, hepatic congestion and biliary cirrhosis, jaundice and pancreatic affections, and that drainage was a necessary and perhaps all-important part in their treatment, and that it could be best done through the cholecyst.

Some surgeons have taken the position that equally effective drainage could be accomplished through the choledochus, but Deaver has well said that such a procedure is not good surgery. First of all, it is not scientific to drain foul and septic bile into the duodenum; not only because it might irritate the bowels, but

1. Read at the Twenty-Second annual meeting of the American Association of Obstetricians and Gynecologists at Fort Wayne, September 21-23, 1909.

because absorption might again take place through its mucous membrane; and further, that the most effective way to get rid of this dangerous material is to discharge it from the body through the gall-bladder at the abdominal wall.

Bland Sutton in a very interesting paper which he read before the surgical section of the British Medical Association in 1907, and which provoked a very spirited and animated discussion, took the position that the gall-bladder is a useless organ without function and a menace to life when diseased, and should always be removed. Three weeks after this discussion there appeared in the *British Medical Journal,* October 26, 1907, an equally important paper by Mayo Robson, who maintained that the gall-bladder served a very useful purpose, and should always be retained unless too seriously damaged, and Deaver, in a masterly article printed in the *American Journal of the Medical Sciences,* April, 1908, argues in a most conclusive manner that the gall-bladder should only be removed under certain grave conditions, or when its walls are so seriously involved and thickened as to render it absolutely useless; not alone because if left, it is the most direct and best drainage channel, but because it is needed by the economy to propel the bile on into the intestines by reason of its tension bulb-like action and in proof of this function, he cites the fact that when the organ has been removed the stump is often found considerably dilated into a sort of diverticulum or secondary gall-bladder.

Another interesting point which adds weight to Deaver's contention, I have observed in my own work—namely, that after the removal of the gall-bladder and also after a simple cholecystostomy, the abdominal incision has broken open some weeks after it had been firmly united, from this great back pressure of bile; and in cases where a patent common duct existed, which was proven by the fact that much free bile was always present in the stools, clear urine was voided and there existed no jaundice even in the conjunctiva.

The old argument that stones recurred in the gall-bladder after drainage had been practised, has been pretty well disproved by the work of Kehr, Mayo, Murphy and Deaver, who combinedly have done over three thousand gall-bladder operations, and in their experience a recurrence of gallstones has been so rare as to make them believe that they were overlooked at the time of operation and were not due to reformation or a recrudescence of the original inflammatory process. Kehr had 2½ per cent. return.

Another objection to the retention of the gall-bladder was the long drain of bile for weeks and sometimes months, which was not only annoying but very distressing, due possibly to the existence of a permanent fistula. Biliary fistulas which require operative measures for their relief are exceedingly rare, owing to a better technic in the performance of cholecystostomy. Heretofore the gallbladder was sewed securely to the abdominal wall, but now the careful surgeon sews his drainage tube into the gall-bladder, and then pursestrings the tube so as to avoid leakage of bile, and in case the gall-bladder falls some distance away from the abdominal wall, he surrounds the tube with a little gauze which is carried through the incision and thus a drainage canal is made by adhesions, and not by permanent fixation of the gall-bladder. Mucus fistulas, on the contrary, require the removal of the gall-bladder, because they have a different pathology, and in order to stop the chronic oozing, the contracted, distorted gall-bladder, which usually has a stone or stricture in the cystic duct, must be ablated.

It is now generally accepted that gall-bladder disease is the result of bacterial invasion, not only from the intestines through the common duct—which is perhaps the most frequent avenue—but also by way of the portal circulation. Organisms of various kinds are absorbed by the tributaries of the portal vein and are carried into the liver substance, then find their way into the gall-bladder, and, under favorable conditions develop a catarrhal or inflammatory process with their associated products. If the infection is severe, an acute cholecystitis results, with the formation of mucus and pus, but not gallstones, and the inflammatory action may be so destructive as to cause ulceration and gangrene and perforation of the bladder wall. Gallstones, however, require for their production a low grade infection with a certain degree of bile stasis, and as a result of this infection we get a proliferative inflammation of the lining membrane of the gall-bladder, and the cells thus produced undergo myelin degeneration, with the formation of cholesterin. The deposition of this cholesterin upon an organic nucleus of blood, pus, mucus, bacteria or any foreign material like silk or catgut, together with accretions of the different bile salts, produces stones of various colors, sizes and composition.

Gallstones usually form in the gall-bladder, but they are occasionally found in the hepatic ducts and bile radicals, and from these different sources find their way into the common duct, where they may remain lodged in its lumen or at the ampulla of Vater, or make their way into the duct of Wirsung, or they may be discharged into the bowel, and are then often collected by sieving the stools. Gallstones may exist and remain in comparative innocence in the gall-bladder and produce very few symptoms other than perhaps the various digestive disturbances peculiar to chronic gall-bladder disease, but if for any reason the bile ducts become more or less obstructed so that the free flow of bile cannot take place, then symptoms varying from slight distress to the greatest agony are complained of. It is believed that attacks of pain are usually the result of the movement of the stones either in the bladder itself or in their passage into the cystic duct, or in their exit through the common duct, together with the coincidental irritation, inflammation and distension of the gall-bladder and the ducts, so that biliary colic or pain is the most common and distressing symptom of calculus disease, but it is also frequently present in gall-bladder disease without stones. The pain is usually referred to the region of the gall-bladder and the pit of the stomach. It often extends to the base of the right scapula or between the scapulæ and runs sometimes into the right shoulder and down the right arm and but rarely to the left side.

Jaundice is a very important symptom, and varies in intensity from a slight staining of the conjunctiva to the most intense bronzing of the skin. It is a variable symptom, and its importance and frequency are variously estimated by different observers. Some say that it exists in only 20 per cent. of the cases, while others claim that it is present at some time in the history of the case very much oftener. It is due to obstruction of the common or hepatic ducts, either from within by stones or stricture, or to adventitious bands and adhesions or growths, angulations and distortions pressing upon their lumen from without. It may also be due, however, to a large stone perhaps impacted in the cystic duct and pressing upon the hepatic at or near its junction to form the common duct. It may be temporary or persistent in character, which depends whether the cause remains stationary or transitory, and the degree is usually in direct proportion to the amount of obstruction. Nevertheless, it is often not present and many capable practitioners have been led into fatal mistakes, and useful lives have been jeopardised and sacrificed by waiting for jaundice before a correct diagnosis was made of calculus disease.

I wish here briefly to give the history of a case which is by no means uncommon, because in my experience, many of my most dangerous operations were performed on persons in whom no jaundice was present, nor could I get any account of its previous existence.

Mrs. L., aged fifty-one; the mother of ten children; a large, stout, fleshy, active woman, whom I saw in consultation with Dr. Hitzel, and diagnosticated gall-bladder disease with gallstones. She had been in bed for two weeks, and her suffering at times was so intense that half a grain of morphia was injected hypodermically. The pains usually came on at night about three or four hours after eating supper. She stated that in none of her confinements did she suffer as in one of these paroxysms. I sent her to the German Hospital, and kept her under observation for a few days. She had no temperature at any time, and twice it was necessary to inject morphine to relieve her pain, but at no time could I elicit pain even upon deep pressure over the gall-bladder, nor could the distented organ be felt through the thick abdominal wall. On the morning of the operation, March 13, 1909, she was slightly jaundiced in the conjunctiva and skin. Upon opening the abdomen along the line of the right rectus muscle, the gall-bladder was seen to be very distented and dark in color. After packing gauze carefully and deeply into the fossa (behind the liver), the gall-bladder was opened, thirty-eight stones were removed, and considerable brownish, ropy fluid. After all the stones were withdrawn by forceps and spoon and the bladder made as clean as possible by very free irrigation, the gauze packs were taken out, the ducts carefully examined, and two large stones were felt deep in the cysticus. An attempt was made to milk them back into the gall-bladder, but this was only partially successful; one was dislodged and delivered through the bladder, but the other was so firmly wedged that every effort to move it was of no avail, so the duct was opened from without and the stone was removed through the opening. It was large, cone-shaped, and evidently filled to distension, the apex of the duct and pressed upon the hepaticus causing the jaundice. The gall-bladder was then separated from the liver and removed after the stump had been carefully tied off and a drainage tube wrapped in gauze was placed to the bottom of the cavity.

The woman reacted well, had an uninterrupted convalescence, and on the eighth day the drain was removed and a smaller piece of tubing was pushed into the wound, as no bile had escaped thus far. On the eighteenth day she complained of great pain over the wound, so hot linseed poultices were applied and frequently changed, and on the following day bile discharged freely from the incision which had opened up; it increased in amount for several days, and then gradually ceased.

In this case the bladder was not seriously crippled, and I removed it simply because I had split the wall so far down, in order to get the stone out of the cystic duct. A stricture, no doubt, existed in the duct, as no bile flowed immediately after the stone was extracted, and it evidently did not yield for many days, because no bile was discharged along the drainage tract until the eighteenth day, when either the cut in the cystic duct opened or the edges of the tied-off gall-bladder separated from the great vis a tergo pressure of the bile current.

Another case upon which I operated on January 29, 1907, had a history of repeated attacks of pain for three years, and then periods of comparative comfort, occasional chills and fever for a few days, but no jaundice. I removed from a healthy-looking bladder four beautifully facetted stones deep down in the cystic duct, which when approximated made one large round concretion the size of a hickory nut. At the time of the operation I thought the cysticus was open and expected that bile would flow freely from the drainage tube, which was pursestringed into the bladder. No bile was ever discharged, but a mucus fistula continued for nine months with periods of acute suffering, hence on September 1, 1907, I operated a second time, removed the gall-bladder and found one small stone deep in the cystic duct, which was overlooked at the first operation. The bladder was small and contracted, but its coats were not specially thickened, and I have no doubt there would have been no trouble occasioned by its retention. It was, however, easier to get the stone when the bladder wall was split down and, therefore, a cholecystectomy was performed. The stump was tied off carefully, but the ligature slipped and a profuse hemorrhage resulted; after some little time the vessel was found, securely occluded, and the wound was quickly closed with drainage. Bile discharged from the wound on the 6th day and continued to flow. The patient did well and has remained in excellent health.

These are the only two cases in a series of twenty-eight in which cholecystectomy was done, and of the remaining twenty-six many were acute empyemas, and six had thickened and contracted gall-bladders but bile oozed through the duct after the stones were removed, so it was thought best to leave the bladder and drain. All did well and have enjoyed good health since the operation.

One case was an inoperable cancer of the gall-bladder in a woman sixty-two years of age. Two patients died, one a stout, young man of forty years of age, whom I had treated for years for syphilis.

I saw him first on March 22, 1906. He was dressed and about his room, but complained of pain and tenderness in the pit of the stomach and was vomiting, which he had attributed to bowling the night before and a little too much beer. I made the diagnosis of gall-bladder disease, and put him to bed. His temperature was 99 4.5. On the following day he was worse, his temperature had risen to 102 2.5 and his pulse was 116; I saw him again in the evening, as I considered him seriously ill, and on the following day his temperature was 104 and pulse 130. There was no jaundice. I took him at once to the German Deaconess's Hospital and operated. I found a very acutely inflamed, enormously distended bladder, with no stones and no adhesions, but there was considerable brownish fluid in the peritoneal cavity; over a pint of pus, mucus and bile was washed out of the bladder, and a tube was sewed into the viscus and the wound closed. The operation was simple and done with dispatch, yet the man died four hours afterward. A postmortem was not permitted.

The second was a woman thirty-eight years of age, the mother of six children, youngest nine years old, whom I operated upon April 10, 1908, at the German Hospital. She had been in bed one week, was jaundiced—although not deeply—and was suffering from pain and tenderness over the pit of the stomach, had nausea and vomiting, and had a temperature of 100 ½ and pulse 90. Upon opening the abdomen, I found the gall-bladder very small and contracted, in a dense bed of adhesions; upon section it was found to contain two small stones and a little mucus. After separating what seemed to be a solid mass of exudate, I came upon the head of the pancreas, which was much enlarged and hard as a stone, probably carcinoma. Bleeding was very free, and the abdomen was closed after leaving a piece of gauze in the wound. The woman died twelve hours after the operation from shock and hemorrhage, and upon postmortem examination, the pancreas was found to be the seat of seirrhous cancer. This fact I did not know until I had done much damage by fruitless tearing of adhesions, because, finding a contracted gall-bladder, I proceeded to expose the ducts.

Courvoirsier's law, which is a very useful guide for the surgeon, would have misled me in this case. He says "that in 84 per cent. of the cases of chronic jaundice, due to obstruction of the common duct, a contraction of the gall-bladder signifies that the obstruction is due to stones; a dilatation of the gall-bladder, that the obstruction is due to causes other than stones." My patient evidently had an old gall-bladder trouble with the production of stones, and a gradual contraction of the organ took place with stricture of the cystic duct and superadded cancer of the pancreas.

In the practice of surgery certain methods of procedure come to us as a result of our own experience, together with the study and careful analysis of the work of other men, and therefore as a general proposition to which, of course, there are some exceptions, I would say that, in my judgment, the gall-bladder should be removed:

1. For acute cholecystitis with gangrene.

2. In chronic cholecystitis when the organ is so thickened and contracted that its future function—if there be one—could not be restored, and where the cystic duct is closed, and, therefore, it could be of no use as a drainage channel.

3. In hydrops where we find a large bladder and cystic duct obstruction.

4. In cancer.

5. In gunshot injuries or perforations from accidental causes, if a partial cholecystectomy is not advisable or possible.

In conclusion, I beg to add that the argument which I have endeavored to make in this paper is that cholecystectomy has been performed altogether too frequently during the past ten years, and that it is often an unnecessary operation. That it has a higher mortality than simple cholecystostomy, that it takes much longer to perform and that the percentage cures of our patients are greater when good drainage is sought for and carried out, and that this drainage is most effectual through the gall-bladder by way of the abdominal wall; and that the post-operative adhesions after cholecystectomy are greater than after cholecystostomy, and as a result, perhaps future suffering. And, finally, if for any reason surgery may again be necessary, the dangers and difficulties are much increased by reason of the absence of the gall-bladder, which is always of service as a landmark to distinguish adjacent structures.

Deaver says:

When in doubt, I say drain rather than remove a gall-bladder which may recover itself; better do this even at the expense of a fistula (mucus) which will call for a second operation, than take out a gall-bladder which may again normally functionate. Better, I say, do two operations in an attempt to save a gall-bladder that may recover itself, and in not a few of these instances save a human life at the same time, than do an operation which will certainly sacrifice the one, if not both.

493 Delaware Avenue.

